# miR-142-5p Disrupts Neuronal Morphogenesis Underlying Porcine Hemagglutinating Encephalomyelitis Virus Infection by Targeting Ulk1

**DOI:** 10.3389/fcimb.2017.00155

**Published:** 2017-05-03

**Authors:** Zi Li, Yungang Lan, Kui Zhao, Xiaoling Lv, Ning Ding, Huijun Lu, Jing Zhang, Huiqing Yue, Junchao Shi, Deguang Song, Feng Gao, Wenqi He

**Affiliations:** ^1^Key Laboratory of Zoonosis, Ministry of Education, College of Veterinary Medicine, Jilin UniversityChangchun, China; ^2^Key Laboratory of Zoonosis, Ministry of Education, Institute of Zoonosis, Jilin UniversityChangchun, China

**Keywords:** Porcine hemagglutinating encephalomyelitis virus, neurotropic virus, miR-142-5p, Ulk1, neuronal morphogenesis

## Abstract

Porcine hemagglutinating encephalomyelitis virus (PHEV) invades the central nervous system (CNS) and causes neurodegenerative disease in suckling piglets, but the understanding of its neuropathogenicity for neurological dysfunction remains limited. Here, we report that miR-142-5p is localized to neurons and negatively regulates neuronal morphogenesis in porcine hemagglutinating encephalomyelitis (PHE). This phenotype was mediated by miR-142-5p inhibition of an mRNA encoding unc-51-like-kinase1 (Ulk1), which controls axon outgrowth and dendrite formation. Modulating miR-142-5p activity by microRNA mimics or inhibitors induced neurodegeneration, including stunted axon elongation, unstable dendritic spine formation, and irregular swelling and disconnection in neurites. Relieving Ulk1 mRNA repression in primary cortical neurons by miR-142-5p antagomirs or replication-deficient adenoviruses encoding Ulk1 (Ad5-Ulk1), which improved rescue of nerve injury, restricted viral replication, and increased survival rate in mice underlying PHEV infection. In contrast, disrupting Ulk1 in RNAi-expressing neurons mostly led to significantly shortened axon elongation and/or an abnormally large number of branched dendrites. Taken together, we demonstrated that the abnormal neuronal morphogenesis underlying PHEV infection was mainly caused by functional mRNA repression of the miR-142-5p target Ulk1. Our data revealed that PHEV adapted to use spatiotemporal control of host microRNAs to invade CNS, and provided new insights into the virus-associated neurological dysfunction microenvironment.

## Introduction

Porcine hemagglutinating encephalomyelitis virus (PHEV) belongs to the order *Nidovirales*, family *Coronaviridae*, and genus *Betacoronavirus*. The virus causes vomiting and wasting disease (VWD) and/or encephalomyelitis in suckling piglets (Vijgen et al., 2006). The virus was first isolated from the brains of suckling pigs with encephalomyelitis in Canada in 1962, after which the disease was reported in Japan, China, Argentina, and other countries (Greig et al., 1962; Mengeling et al., 1972; Hirai et al., 1974; Pensaert and Callebaut, 1974; Sasseville et al., 2001; Quiroga et al., 2008; Gao et al., 2011; Dong et al., 2014). The mortality rate of PHEV-infected piglets under 3 weeks of age is almost 100%, whereas older pigs generally exhibit subclinical signs of infection. PHEV is a highly neurovirulent virus that invades the central nervous system (CNS) via the peripheral nerves, specifically focusing on microtubules and the intermediate filaments of nerve cells (Yagami et al., 1993; Hirano et al., 1995, 2004; Lyi et al., 2014; Foo and Chee, 2015). Thus far, the mechanism for the neurotropism of PHEV remains unclear. Given that PHEV antigen-positive nerve cells are distributed widely in the cerebral cortex (Hirano et al., 1998), a clarification of the pathogenesis in cortical neurons is expected to aid in understanding PHEV infectivity and propagation in the CNS.

The discovery of microRNAs has greatly expanded our understanding of the cellular mechanism that regulates gene expression via base pairing with mRNAs (Ambros, 2004; Bartel, 2004). Studies on microRNA:mRNA interaction networks have uncovered a notable role of the microRNA system in the pathogenesis of RNA viruses: many viruses exploit their host pathways by destroying, boosting, or hijacking microRNAs to benefit the viral life cycle (Linnstaedt et al., 2010; Guo et al., 2014; Guo and Steitz, 2014; Luna et al., 2015). For example, Trobaugh et al. (2014) noted that the North American eastern equine encephalitis virus (EEEV) adapts to use the antiviral properties of vertebrate miR-142-3p to limit replication in particular cell types, a restriction that can lead to exacerbation of disease severity. As for PHEV, the single-stranded RNA virus causes nervous system dysfunction in suckling piglets or mice, and the infection locus encodes huge differentially expressed microRNAs. Given that, biochemical and genetic studies have revealed that dysregulation of microRNA expressed in neurological dysfunction and virus progression (Yu et al., 2015; Kadri et al., 2016), and that the microRNA is significantly altered in a subset of patients with neuroAIDS, traumatic brain injury (TBI), epilepsy (Winkler et al., 2012; Wang et al., 2015). Investigating the microRNA profiling underlying PHEV infection, and determining the role of microRNA system in PHE may provide insight into temporal, geographical, and individual host variations in potential CNS injury diseases. Of these microRNAs identified by microarray, we found that miR-142-5p was highly induced after PHEV infection. Nevertheless, even if miR-142-5p might control translation of nerve injury associated mRNAs in PHE have yet to be investigated.

In the current study, we discovered that the upregulation of miR-142-5p (*Accession number: MIMAT0000154*) severely stunted neuronal morphogenesis during PHEV infection by targeting the unc-51-like-kinase1 (Ulk1, *NCBI Reference Sequence: NM_009469.3*) mRNA 3′ untranslated region (3′UTR), which suggested that PHEV largely exploited spatiotemporal control of host microRNAs for neurological disorders. Expression analysis also supported a role for miR-142-5p in the early stages of neuronal development and neurite formation. Further characterization of the downstream mRNAs targeted by miR-142-5p showed that Ulk1, a serine/threonine kinase, is one of the prospective PHEV-regulated mRNAs that are relevant to nerve dysfunction. Thus, we proposed that miR-142-5p-mediated Ulk1 repression might lead to a breakthrough in the understanding of the mechanism of neurological dysfunction induced by PHEV. The results of this study should enhance our collective understanding of how these neurotropic viruses disturb the CNS through post-transcriptional events.

## Materials and methods

### Ethics statement

This study was carried out in accordance with the recommendations of the Council for International Organization of Medical Sciences on Animal Experimentation (World Health Organization, Geneva, Switzerland). The protocol was approved by the Animal Welfare Ethical Committee of the College of Veterinary Medicine, Jilin University, China.

### Animal protocols

Briefly, 3-week-old BALB/c mice (male) were intranasally inoculated with 0.1 mL PHEV (10^4.45^ 50% tissue culture infectious doses [TCID_50_]/0.1 mL, GenBank: AY078417.1) or equal amounts of phosphate-buffered saline (PBS, 1 M, pH 7.4). At 24 h after PHEV infection, the inoculated mice were anesthetized with 3.5% chloral hydrate (1.0 mL/100 g; Sigma, USA), and 10 nmoL miR-142-5p antagomirs or negative control antagomirs were then positioned in the cerebral cortex with a stereotaxic apparatus (David Kopf Instruments, Tujunga, CA, U.S.A) using previously described methods (Kirby et al., 2012; Wu et al., 2012). The antagomirs used here, are a class of chemically engineered and modified oligonucleotides that prevent other molecules from binding to a desired site on an mRNA molecule, and purchased from RiboBio in China. Mice were sacrificed at various timepoints, and the cerebral cortices of PHEV- or control-infected mice were dissected for the following study.

### Primary neuron culture and transfection

Primary cortical neurons from embryonic day 18 (E18) Sprague–Dawley rats were prepared as described previously (Schratt et al., 2004). The neurons were maintained in Neurobasal plus B27 supplement (Invitrogen, Gaithersburg, MD) on plates coated with poly-D-lysine (Sigma, St. Louis, MO). Replication-deficient adenoviruses encoding Ulk1 (Ad5-Ulk1) were generated by homologous recombination. Ad5-green fluorescence protein (Ad5-GFP) was used as a parallel control during gene transfection. Neuronal transfections were performed with Lipofectamine RNAiMAX reagent (Life Technologies, Rockville, MD) as previously described (Zhao et al., 2008; Kim et al., 2014). For both gain-of-function and loss-of-function study, 5~100 nM miR-142-5p mimics or 50~200 nM inhibitors were transfected into neurons using X-tremeGENE HP DNA Transfection Reagent (Roche, Sweden), and these oligonucleotides were designed as follows: mimic sense primer, 5′CAUAAAGUAGAAAGCACUACU3′; mimic anti-sense primer, 3′ GTAUUUCAUCUUUCGUGAUGA5′; inhibitor oligonucleotides, 5′mAmGmUmAmGmUmGmCmUmUmUmCmUmAmCmUmUmUmAmTmG3′. Methylate-modified bonds were indicated as m.

### Quantitative RT-PCR

For quantitative RT-PCR, total RNA was extracted using Trizol and miRNA was purificated by the miRNeasy Mini Kit (Qiagen, Germany), and all was quantified using a SmartSpec™ plus Spectrophotometer (BIO-RAD, USA). Reverse transcription was performed using Bulge-Loop microRNA-specific reverse transcription-primers (RiboBio, Guangzhou, China) and PrimeScript Reverse Transcriptase (Takara, Japan). Quantitative PCR reactions were conducted with SYBR® Premix Ex Taq™ II (Takara, Japan) to analyze Ulk1 expression with GAPDH as a normalization control. MiR-142-5p level was detected using Bulge-Loop primers (RiboBio) on a CFX96 Touch™ Real-Time PCR Detection System (Bio-Rad, USA), and small nuclear RNA U6 as a normalization control. The primers for U6 and GAPDH were designed as follows: U6 sense primer, 5′CTCGCT -TCGGCAGCACA 3′; U6 anti-sense primer, 5′AACGCTTCACGAAYYY GCGT 3′; GAPDH sense primer, 5′CTCAACTACATGGTCTACATGTTC3′; GAPDH anti-sense primer, 5′ATTTGATGTTAGTGGGGTCTCGCTC3′. The quantitative RT-PCR reaction conditions: pre-degeneration at 95°C for 3 min, denaturation at 95°C for 30 s, annealing at 60°C for 30 s, extension at 72°C for 30 s with a total of 40 cycles.

### Western blotting

Primary cortical neuron lysate or freshly isolated cerebral cortex lysate was diluted in RIPA lysis buffer on ice. Subsequently, SDS-PAGE was performed, and the lysate samples were electrotransferred to 0.22 μm polyvinylidene difluoride membranes using the Bio-Rad wet transfer system. And then, the membranes were blocked overnight at 4°C with 5% non-fat dry milk in PBS and immunoblotted with antibodies against Ulk1 (Cell Signaling Technologies, USA, 1:1,000), β-actin (Proteintech, USA, 1:2,000) and PHEV (provided by our laboratory, 1:500) at 4°C overnight. Next, incubation with horseradish peroxidase (HRP)-conjugated secondary anti-rabbit or anti-mouse IgG antibodies (Proteintech, USA) was performed for 1 h at 37°C.

### Immunocytochemistry (ICC)

Primary cortical neurons were fixed in 4% paraformaldehyde (Fisher Scientific, Waltham, MA), permeabilized with 0.2% Triton X-100 and blocked in bovine serum albumin (BSA). Immunostaining was performed overnight with microtubule-associated protein 2 (MAP2) (D5G1) XP® rabbit mAb (Cell Signaling Technologies, USA), ULK1 (D8H5) rabbit mAb, and/or PHEV mouse mAb (provided by our laboratory) in 1% BSA at 4°C. Alexa Fluor 568- or Alexa Fluor 488-conjugated anti-mouse or anti-rabbit secondary antibodies (Invitrogen, 1:1,000) were applied for 1 h at room temperature. After counterstaining with 1 μg/mL Hoechst 33342 (Sigma) for 2 min, coverslips were mounted with ProLong Gold Antifade Reagent (Life Technologies), and the slides were observed under a confocal microscope (FV10-ASW 3.0, Olympus Europa Holding GmbH).

### Immunofluorescence staining

For immunofluorescence staining, fresh brain tissues frozen in liquid nitrogen and embedded in OCT compound were cut into cryostat sections (8 μm) and mounted on Superfrost Plus slides. Before staining, the slides were warmed at room temperature for 30 min and were then immersed in blocking buffer (10% serum and 0.1% Triton X-100 in PBS) for 1 h at room temperature. Slides were then treated with primary antibodies overnight at 4°C and subsequently incubated with secondary antibodies conjugated with either Alexa 488 or Alexa 594 for 30 min at room temperature. After counterstaining the samples, the coverslips were mounted and viewed under a confocal microscope.

### *In situ* hybridization(ISH)

*In situ* hybridization of endogenous mRNAs and microRNAs was performed as described (Wibrand et al., 2010). Briefly, tissue sections and primary cortical neurons were fixed with 4% paraformaldehyde/DEPC-PBS. After pre-hybridization in hybridization buffer at 55°C for 2 h, hybridization with digoxigenin (DIG)-labeled mRNA probes or biotin-labeled microRNA fluorescence *in situ* hybridization (FISH) probes (EXONBIO, Guangzhou, China) was performed at 42°C overnight. Subsequently, blocking reagent was applied, followed by incubation with an aminomethylcoumarin (AMCA)-conjugated anti-DIG or rhodamine-conjugated anti-biotin antibody (EXONBIO, Guangzhou, China) at 37°C for 30 min together with a MAP2 rabbit mAb (in the dark). After counterstaining the samples with 4',6-Diamidino-2-phenylindole (DAPI)-Antifade at room temperature for 20 min, the slides were examined under a fluorescence microscope with a proper filter set.

### Luciferase reporter assay

The rat Ulk1 3′UTR was amplified by RT-PCR from mouse brain cDNA (P15). Mutation of the miR-142-5p binding site was achieved using the Multisite-Quickchange kit (Stratagene, USA) according to the manufacturer's protocol. To further confirm the regulation of Ulk1 by miR-142-5p, the luciferase pmirGLO reporter (Promega, Madison, USA) was constructed and then confirmed by sequencing. Luciferase activity was detected 48 h after the co-transfection of the luciferase construct (with either wild-type or mutant-type miR-142-5p binding sites), the miR mimics/inhibitors or control (RiboBio, Guangzhou, China), and a Renilla luciferase vector in HEK293T cells. The Dual-Luciferase Reporter Assay System (Promega, Madison, USA) was used to quantify the effects of a miR-142-5p interaction with the Ulk1 3′UTR.

### Electrophoretic mobility shift assay

The validation of microRNA-mRNA interactions was performed using the Molecular Probes' fluorescence-based Electrophoretic Mobility Shift Assay (EMSA) Kit (Invitrogen, Gaithersburg, MD) according to the manufacturer's protocol. For the binding assays, the following RNA and DNA oligonucleotides (Sigma-Aldrich, Madrid, Spain) were designed and used: miR-142, an RNA sequence corresponding to the mature form of miR-142-5p; Ulk1-UTR, a 23-mer RNA sequence for the 3′UTR corresponding to Ulk1 with the target site for miR-142-5p; anti-miR-142, a modified antisense oligodeoxynucleotide complementary to the sequence of the mature form of miR-142-5p; and anti-miR-142MIS, an antisense oligodeoxynucleotide containing 11 mismatches compared to anti-miR-142. All RNA and DNA oligonucleotides were purchased from Sigma-Aldrich (Madrid, Spain), and their specific sequences are listed in the Table S1. The corresponding RNA or DNA molecules were incubated in binding buffer (750 mM KCl, 0.5 mM dithiothreitol, 0.5 mM EDTA, 50 mM Tris, pH 7.4) for 30 min at 37°C, and the reaction products were then separated on a 10% non-denaturing polyacrylamide gel. After staining the gel with SYBR® Green solution for 20 min in the dark, it was photographed using 300 nm UV transillumination.

### RNA interference

Neurons were transfected with 20, 50, and 100 nM siRNA directed against Ulk1 (20 μM, RiboBio, Guangzhou, China) using Lipofectamine RNAiMAX reagent (Life Technologies, Rockville, MD) at 10 DIV. Sequences of all targeting oligonucleotides are in the Table S2. Neurons were cultured for additional 2–3 days at 37°C, and the silence effect of siRNA treatment on Ulk1 expression was determined by western blotting. Subsequently, neurons were subjected to further treatments, and harvested for immunofluorescence staining.

### Image and statistical analyses

For outgrowth and length analyses, 20 sections per coverslip and more than 50 cells were quantified and analyzed using the ImageJ plugin Neuron J. The lengths and numbers of neurites were presented as relative values compared to the control group and were compared per condition for a total of three independent experiments. Statistical analysis was performed with either Student's *t*-test or one-way ANOVA in GraphPad Prism version 5 software. A P value less than 0.05 was defined as the threshold for statistical significance.

## Results

### miR-142-5p expression was induced in response to PHEV infection

**Figure 1:**
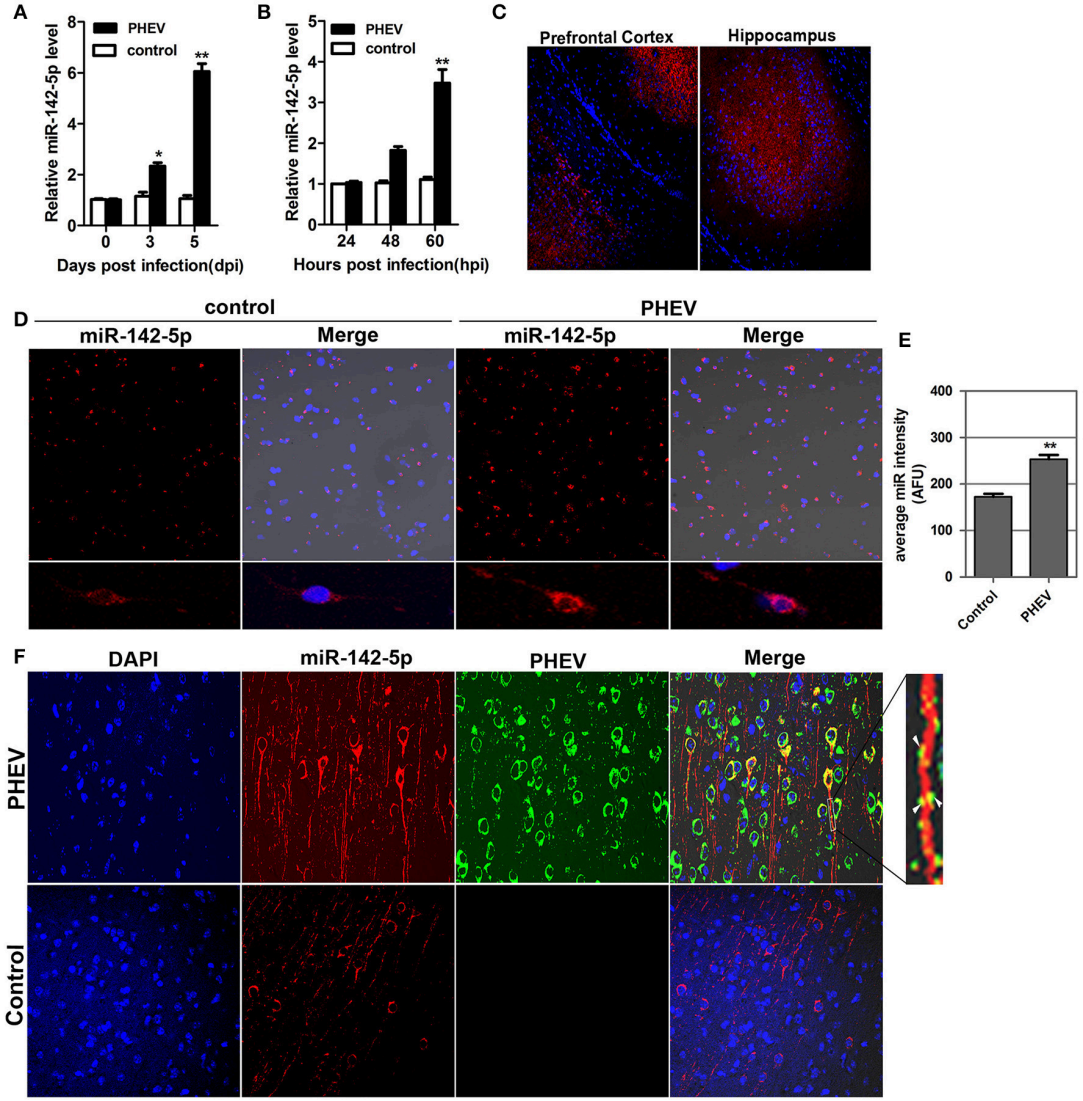
**Validation of miR-142-5p expression ***in vivo*** and ***in vitro***. (A)** qRT-PCR analysis of miR-142-5p expression in the cerebral cortex of mice infected, or not infected, with PHEV. **(B)** qRT-PCR analysis of miR-142-5p expression in primary cortical neurons. miR-142-5p expression normalized to U6 was examined, and the data are presented as the means ± SEM (*n* = 6). **(C)** ISH analysis in frozen section showed miR-142-5p RNA expression (red) in the prefrontal cortex and hippocampus. **(D)** ISH analysis in primary cortical neurons showed miR-142-5p (red) was up-regulated by PHEV. **(E)** Quantitative analyses of miR-142-5p RNA in primary cortical neurons infected, or not infected, with PHEV. The y-axis is the average miR-142-5p intensity using arbitrary fluorescence unit (AFU), **P < 0.01. **(F)** Two-color immunofluorescence staining of miR-142-5p RNA probe (red) and anti-PHEV (green) in cortical neurons in mice. Partial green staining co-localized with red staining, and several dendrite protrusions appear to be yellow (Arrow).

In our previous study, a microRNA array was performed to identify the microRNAs involved in neurological dysfunction as a result of PHEV infection (unpublished data). Of the up-regulated microRNAs, miR-142-5p, the expression of which increased over 5-fold, was selected for confirmation by quantitative real-time polymerase chain reaction (qRT-PCR). miR-142-5p expression was time-dependently upregulated in response to PHEV infection in mice (Figure 1A), consistent with the microarray analysis. Meanwhile, PHEV-infected primary cortical neurons also induced a significant increase in miR-142-5p RNA expression (Figure 1B). We used an *in situ hybridization* (ISH) protocol to examine the localization of miR-142-5p RNA and found widespread expression of microRNAs in the cerebral cortexes of the mice, especially near the prefrontal cortex and hippocampus (Figure 1C). Hybridization with the miR-142-5p-specific probe in primary cortical neurons revealed that miR-142-5p localization occurred in a punctuate pattern along the axon shaft and within dendrite protrusions, especially at branching points (Figure 1D). Also, the signal intensities confirmed significantly higher levels of the miR-142-5p in PHEV-infected neurons (Figure 1E). Some co-localization was observed in the axon and dendrite through two-color immunofluorescence staining of both miR-142-5p and PHEV (Figure 1F). These data suggest that miR-142-5p RNA expression was significantly up-regulated in neurons infected with PHEV. Moreover, the co-localization of miR-142-5p and PHEV within neurites suggested that there is a possible functional role for this microRNA in PHEV-induced nerve cell injury.

### miR-142-5p inhibitors restricted PHEV replication *In vitro*

**Figure 2:**
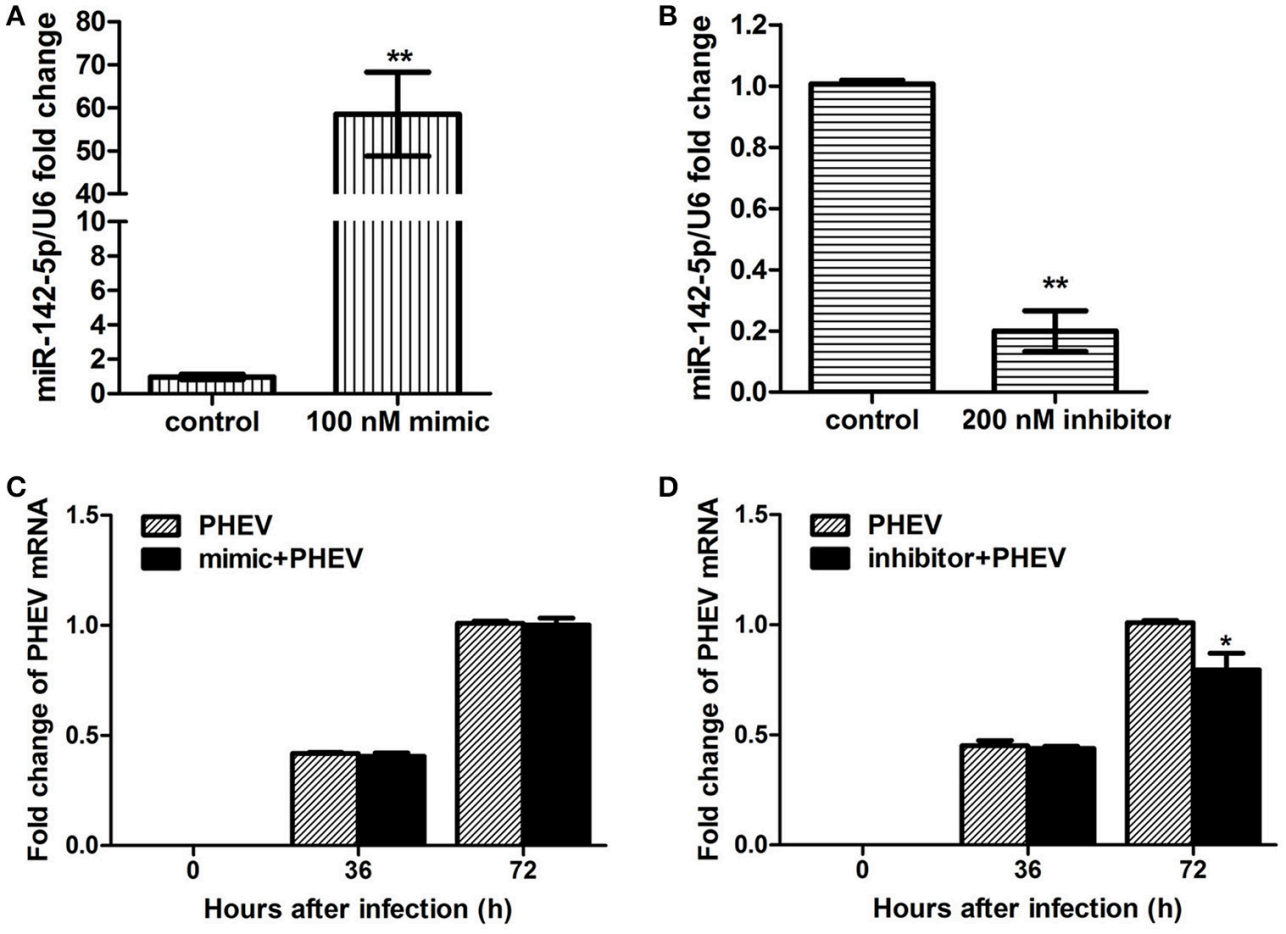
**Impact of miR-142-5p mimics and inhibitors on PHEV replication**. Neurons were cultured for a total of 10 days (10DIV), transfected at 7 DIV, PHEV-infected at 8 DIV, and then cultured for 48 h *in vitro*. **(A)** Mimics were introduced to permit efficient upregulation of miR-142-5p levels in neurons. **(B)** Inhibitors were introduced to permit efficient downregulation of miR-142-5p levels in neurons. **(C)** miR-142-5p mimics (100 nM) were not effective in restricting PHEV replication in neurons. **(D)** miR-142-5p inhibitors (200 nM) significantly decreased viral replication at 72 h post-infection (hpi) (*P < 0.05, **P < 0.01).

To achieve efficient overexpression of exogenous miR-142-5p, microRNA mimics were transfected into primary cortical neurons. Alternatively, miR-142-5p function was suppressed by transfecting a microRNA inhibitor that interfered with endogenous miR-142-5p activity in a sequence-specific manner (Hutvagner et al., 2004). All transfection efficiency was first tested in neurons, and we found that 100 nM of the mimics greatly enhanced exogenous miR-142-5p expression (Figure 2A), whereas 200 nM of the inhibitors led to a statistically significant decrease compared to the negative group (Figure 2B). The transfected neurons were incubated with PHEV 24 h later, and we found that the mimics were not significantly functional in PHEV-infected neurons for all time points (Figure 2C), whereas PHEV replication was restricted in the inhibitor-transfected neurons (Figure 2D). These data indicated that miR-142-5p inhibitors contribute to PHEV replication restriction.

### mir-142-5p regulated neuron morphology

**Figure 3:**
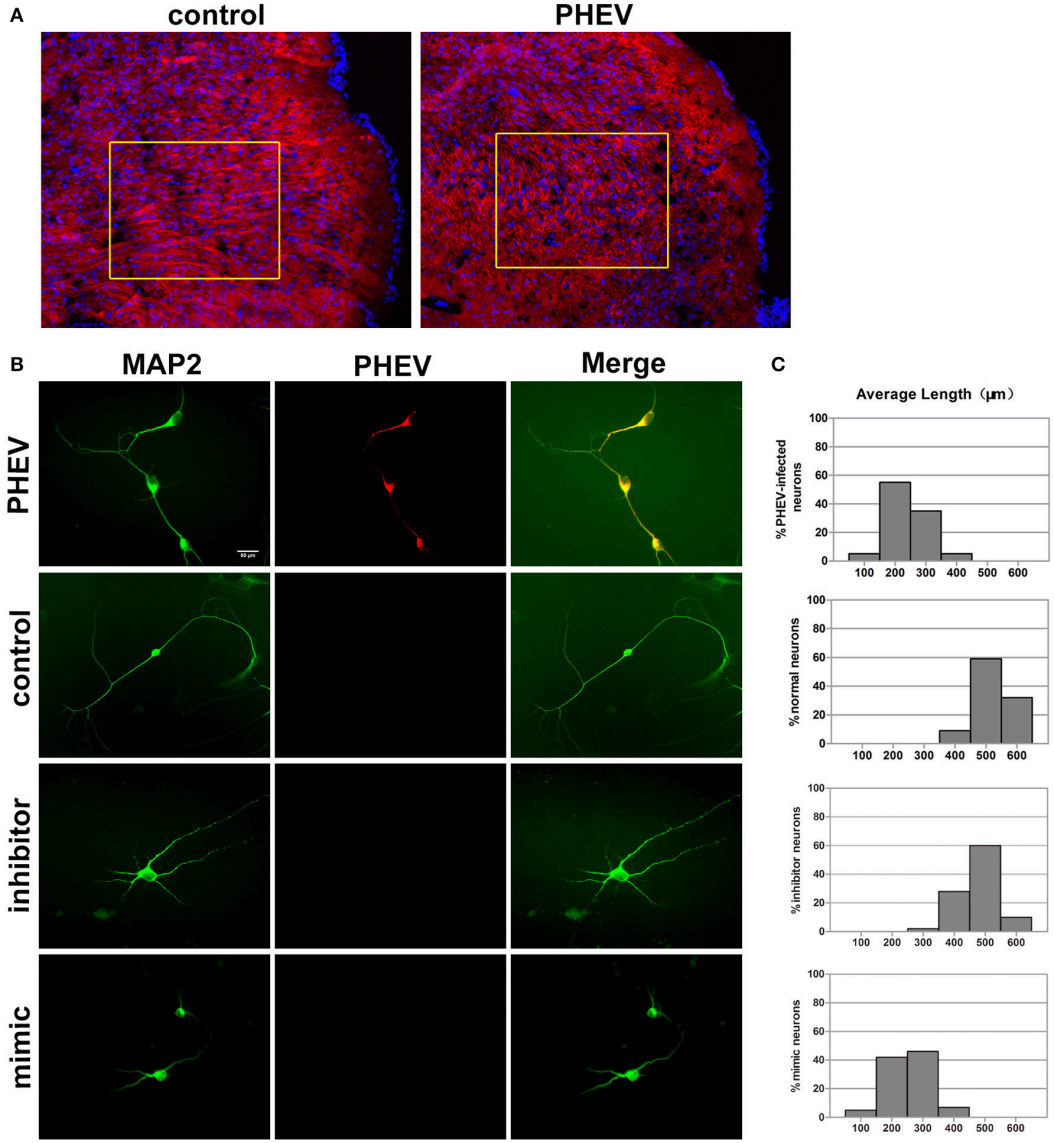
**miR-142-5p negatively regulated axon elongation in cortical neurons. (A)** Two-color immunofluorescence staining of anti-MAP2 (red) and Hoechst 33342 (blue) on a portion of the cerebral cortex in frozen mouse brain sections. Most red staining of the axonal flow of PHEV-infected mice shows spreading of the disorder (inside the box). **(B)** PHEV-infected neurons grew significantly shortened axons (upper panel), while normal control neurons showed bipolar morphology, with two main long axons. Neurons transfected with miR-142-5p inhibitors had similar morphologies as normal neurons. All mimic-transfected neurons had shorter axons. Antibody straining of MAP2 (green) identified dendrites. **(C)** Histograms represent the percentages of neurons with different lengths after PHEV infection, mimics/inhibitors transfection, and control treatment. The y-axis represents the percentage of neurons, and the x-axis represents the length of axons grouped into five columns.

In previous studies, PHEV has been suggested to spread within and among nerve cells via microtubules and intermediate filaments (Hara et al., 2009). Unexpectedly, we found that the spreading, stretching and arranging of neurons in PHEV-infected mice were disordered (Figure 3A). Moreover, primary cortical neurons had significantly shortened axon elongations upon PHEV infection at 8 days post-infection (dpi) (Figure 3B, upper panels). The presence of miR-142-5p in axons suggested that this microRNA might be involved in the regulation of embryonic cortical neuron morphology in response to PHEV *in vivo* and *in vitro*. To investigate this possible function, we examined the effects of modulating miR-142-5p activity on cortical neuron morphology. Representative images showed that both control neurons and inhibitor-transfected neurons (Figure 3B) mostly grew with two main axons that extended over a long distance, but significantly decreased axon elongation in neurons was detected when miR-142-5p was overexpressed (Figure 3B, lower panels). We also measured the average length of the longest axon for each of the transfection categories, and quantitative analyses of these phenotypes were summarized in histograms (Figure 3C). These findings suggested that miR-142-5p overexpression negatively affect cortical neuron development and morphogenesis. We concluded that miR-142-5p acted as a negative regulator of axon elongation in primary cortical neurons and was involved in the regulation of axon development and/or functional responses to PHEV.

### Algorithms predicted target mRNAs for the miR-142-5p

**Figure 4:**
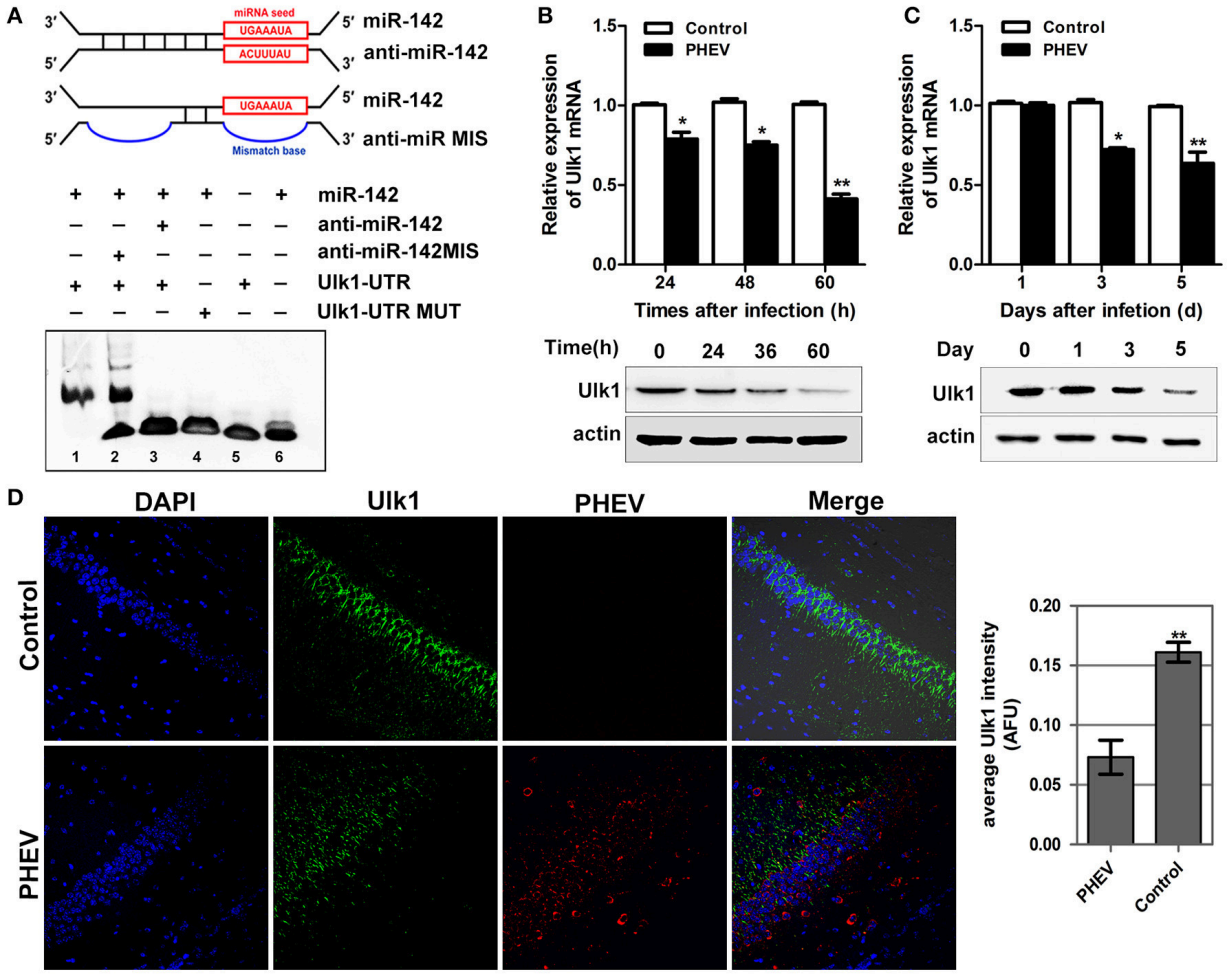
**Ulk1 expression level was down-regulated after PHEV infection. (A)** For the binding assays, RNA and DNA oligonucleotides were designed according to the model and corresponded to the incubation of the probes for EMSA on a non-denaturing gel. Lane 1 represented the binding and competition between miR-142-5p and the Ulk1 3'UTR. **(B)** Ulk1 expression was inhibited upon PHEV stimulation in primary neurons. **(C)** Ulk1 mRNA and protein levels were gradually decreased in the cerebral cortexes of PHEV-infected mice. **(D)** Representative immunocytochemistry results for Ulk1 expression (green) in the cerebral cortex of PHEV-infected or healthy mice. Quantitative analyses of the PHEV staining were list on the right. Error bars for all experiments represent the geometric means ± s.d. Asterisks indicate differences that were statistically significant (**P < 0.01).

To gain insight into the mechanisms by which miR-142-5p regulates axon morphology during PHEV infection, we sought to identify miR-142-5p target mRNAs. Potential targets of miR-142-5p were predicted using the microRNA target prediction databases, including TargetScan, miRBase, and and miRwalk. For this analysis, we focused on a set of 10 genes that we recently identified in a screen for mRNAs in which translation was reduced in neurons upon treatment with PHEV (Lan et al., 2014). Among these mRNAs, Ulk1 was of particular interest and was found to contain conserved 3'UTR sequence elements that were partially complementary to mouse miR-142-5p. Using an electrophoretic mobility shift assay (EMSA), we demonstrated that Ulk1 mRNA and miR-142-5p interacted *in vitro* (Figure 4A). qRT-PCR and Western blotting analysis showed that Ulk1 expression levels were all down-regulated reciprocally with miR-142-5p in the primary cortical neurons after PHEV treatment, and that Ulk1 mRNA expression was nearly 1.2-fold lower compared to the expression in normal neurons within 60 hpi (Figure 4B). Consistent results were observed in mice infected with PHEV, and Ulk1 expression was reduced to the lowest level in the cerebral cortex of infected mice at 5 dpi (Figure 4C). Moreover, an immunocytochemistry assay further confirmed that Ulk1 expression decreased in the injured brain tissue compared to the normal brain tissue (Figure 4D). Therefore, we hypothesized that Ulk1 mRNA was a putative miR-142-5p target gene and may be involved in PHEV-induced neuronal damage.

### mir-142-5p inhibited translation of Ulk1 mRNA

**Figure 5:**
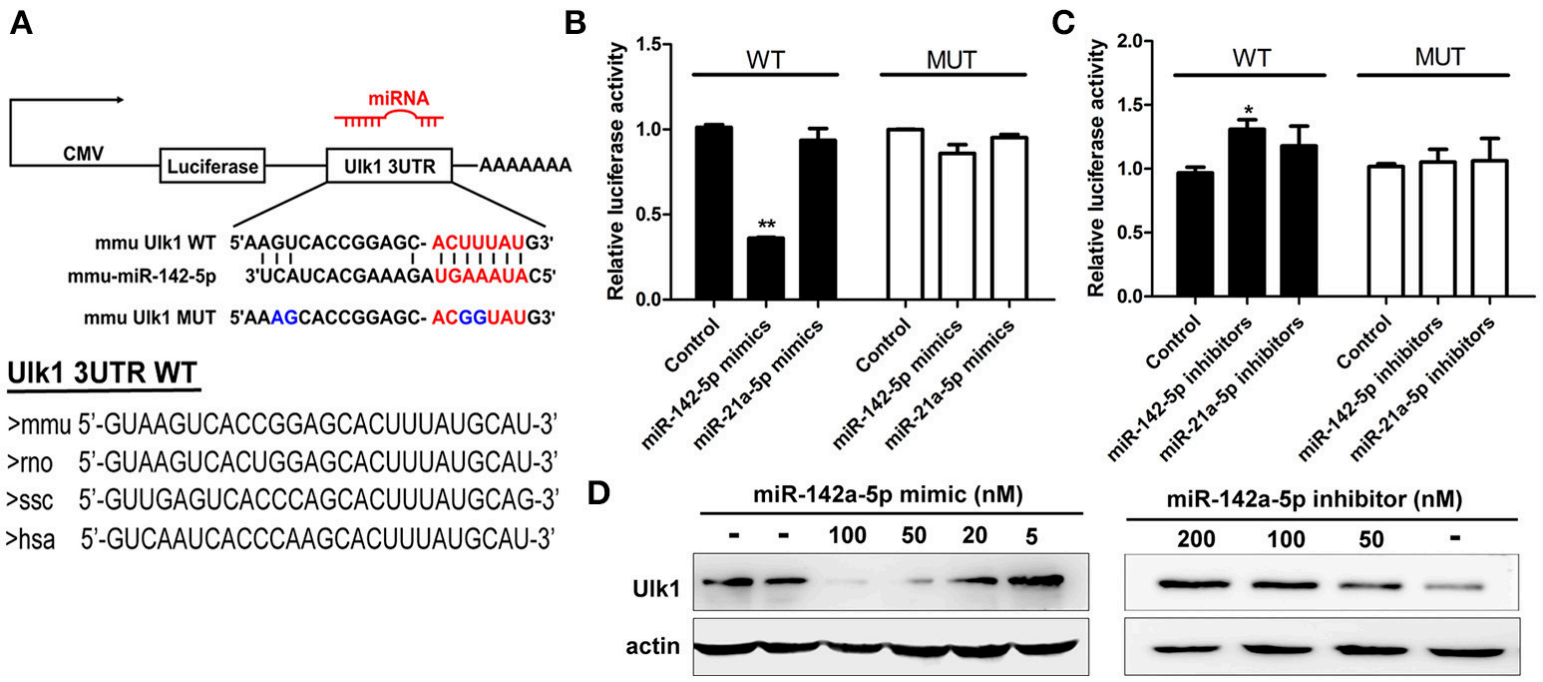
**miR-142-5p negatively regulated Ulk1 expression. (A)** Schematic drawing of the putative binding sites or mutations of miR-142-5p binding-sites in Ulk1 mRNA 3'UTR. Sequence of the mmu MUT-Ulk1 3'UTR containing mutations (blue) in the miR-142-5p binding site was listed below. Lower panel: Sequence conservation of miR-142-5p binding site within Ulk1 3'UTR of mouse (mmu), rat (rno), pig (ssc), and human (hsa). **(B)** Luciferase activity of wild-type (black bars) or mutant-type (white bars) Ulk1 3'UTR reporter genes in the absence (control) or presence of the indicated mimics (50 nM). **(C)** Luciferase activity of reporter genes described in **(B)** in the presence of the indicated inhibitors (200 nM). **(D)** Western blotting analysis of Ulk1 expression in lysates from primary cortical neurons transfected with miR-142-5p mimics/inhibitors. Data represent the means from three independent experiments±SD, Paired Student's *t*-test, *P < 0.05, **P < 0.01.

To confirm that miR-142-5p bound to the Ulk1 mRNA 3′UTR, we performed a luciferase reporter assay, which involved the wild-type or mutant-type Ulk1 3′UTR (Figure 5A). Transfection with miR-142-5p mimics in neurons specifically decreased the activity of a luciferase reporter gene fused to the wild-type Ulk1 3′UTR, whereas overexpression of the unrelated miR-21a-5p microRNA had no significant effect on the expression of this reporter construct (Figure 5B, black bars). The effect of miR-142-5p on translation of the luciferase mRNA was dependent on the presence of the miR-142-5p cognate binding site within the 3′UTR, and expression of a luciferase reporter containing the mutant-type Ulk1 3'UTR was unaffected by the presence of exogenous miR-142-5p (Figure 5B, white bars). In contrast, miR-142-5p inhibitors led to a statistically increased expression of wild-type Ulk1 3'UTR reporter (Figure 5C, black bars), but less effect on the mutant one (Figure 5C, white bars). Moreover, analysis of Ulk1 expression in lysates from miR-142-5p mimics transfected primary cortical neurons showed a dose-dependent decrease in the level of endogenous Ulk1 protein, whereas delivery of its inhibitor led to an increase in protein expression (Figure 5D). Further investigation determined that Ulk1 level was inhibited *in vivo* after the miR-142-5p antagomir was injected into the brains of mice with a stereotaxic apparatus (**Figure 9A**, left panel). Taken together, these data indicated that miR-142-5p inhibited Ulk1 expression by binding to a single site present in the Ulk1 mRNA 3′UTR, and Ulk1 was a nerve injury associated mRNA down-regulated by miR-142-5p in PHE.

### Ulk1 functions in axonal growth and morphogenesis

**Figure 6:**
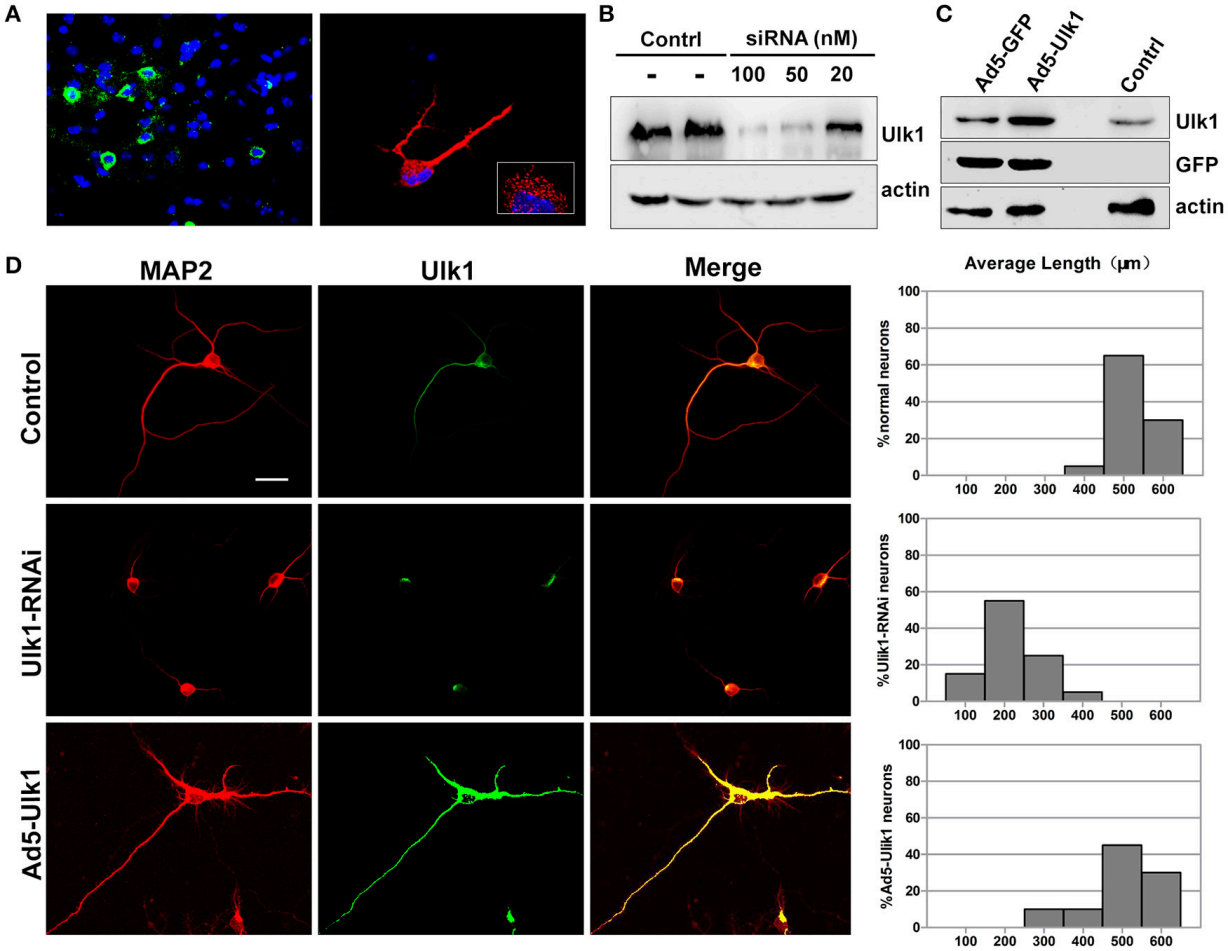
**Ulk1 plays a key role in axon elongation in neurons. (A)** Hybridization with the Ulk1 probe revealed that Ulk1 was present in soma, neurites and growth cones within punctate structures in primary cortical neurons (red) and in mouse cortical neurons (green). The boxed inset in the lower-right panel is shown at greater magnification in the right panel. **(B)** The primary cortical neurons were transfected with siRNA at a concentration of 20, 50, and 100 nM. Forty-eight h after transfection, the cells were harvested for analysis of Western blot, and results revealed that 100 nM siRNA effectively knocked down Ulk1 protein expression in primary cortical neurons. **(C)** The primary cortical neurons were transfected with Ad5-Ulk1 or Ad5-GFP at a MOI of 100. Forty-eight h after transfection, the cells were harvested for analysis of Western blot. **(D)** Antibody staining of RNAi- and Ad5-Ulk1- expressing primary cortical neurons showed a reduction of axon length compared to the controls. Scale bars are 50 μm in the image. In each histogram, the y-axis represents the percentage of neurons, and the x-axis represents the length of the axons grouped into five columns.

*In situ hybridization* of brain tissue sections and primary cortical neurons showed that Ulk1 had a punctate distribution and was localized along the axon shaft and within growth cones (Figure 6A). Because the Ulk1 mRNAs were suppressed upon PHEV infection in neurons, it is possible that Ulk1 is involved in axonal growth and morphogenesis. To confirm a specific role for the Ulk1 proteins, we performed RNAi and overexpression analyses in primary cortical neurons. The siRNA construct directed against Ulk1 is outlined in the Table S2, and its efficiency and specificity in the knockdown of target proteins was first confirmed by Western blot (Figure 6B). To investigate the effect of overexpression of Ulk1, replication-deficient adenoviruses encoding Ulk1 (Ad5-Ulk1) were generated by homologous recombination, and it significantly increased the proportion of Ulk1 at a multiplicity of infection (MOI) of 100 compared with the untreated cells (Figure 6C). Antibody staining of primary cortical neurons revealed that disrupting Ulk1 in RNAi-expressing neurons mostly led to significantly shortened axon elongation and/or an abnormally large number of branched dendrites. In contrast, Ad5-Ulk1 infection resulted in excessive axon arborization and axon elongation, while the control neurons mostly grew in a normal mode with a main axon that extended over a long distance (Figure 6D). Quantitative analyses of these phenotypes were described and summarized in a histogram, and the analyses indicated that Ulk1-RNAi-expressing neurons were less than approximately half the length of controls. Taken together, our data indicated that Ulk1 played a key role in neuronal morphogenesis and the dysfunctional Ulk1 disturbed axonal elongation in primary cortical neurons.

### miR-142-5p-mediated Ulk1 repression controls axon elongation underlying PHEV infection

**Figure 7:**
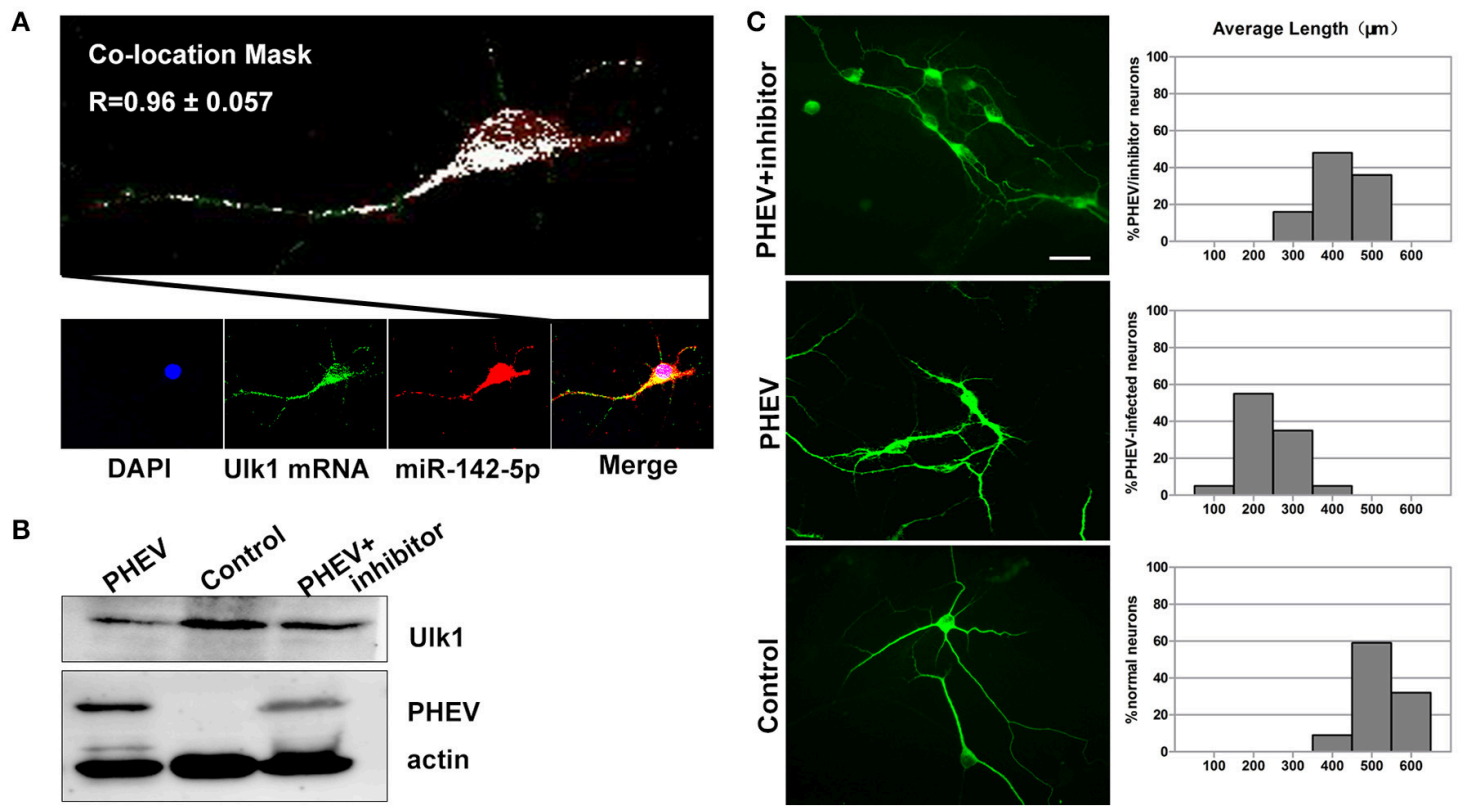
**Enhanced Ulk1 by miR-142-5p inhibitors rescued axon elongation. (A)** Co-localization assay of miR-142-5p (red) and Ulk1 mRNA (green) in primary cortical by Image J software. Pearson's *R* = 0.96 ± 0.057. **(B)** The PHEV-infected neurons were transfected with miR-142-5p inhibitors at a concentration of 200 nM. Forty-eight h after transfection, the cells were harvested for analysis of Western blot. **(C)** The PHEV infected neurons were transfected with miR-142-5p inhibitors at a concentration of 200 nM for 48 h. Antibody straining showed the infected neurons transfected with inhibitors did not have restricted axon elongation. Bar graph: quantification of neurons with different axon lengths. Data represent the means of *n* = 50 cells per condition counted in triplicate ± s.d. Scale bars are 50 μm in the image.

Based on previous findings, we further explored the basic mechanisms underlying the axon morphology changes caused by PHEV infection. Co-expression of miR-142-5p and Ulk1 was found in the neurite shifts and/or growth cones with a prominent signal in primary neurons (Figure 7A), which provided evidence for miR-142-5p-mediated Ulk1 repression as a possible explanation for the reduction in axonal elongation. To substantiate this hypothesis, miR-142-5p inhibitors were used to promoting Ulk1 expression in PHEV-infected neurons (Figure 7B), and we found that miR-142-5p inhibitors efficiently rescued the shortened axon elongations caused by loss function of Ulk1 (Figure 7C). Thus, we concluded that Ulk1 mRNA is a downstream element of miR-142-5p in the regulation of axon outgrowth, and that inhibiting miR-142-5p expression in neurons should be able to improve the axon defect caused by PHEV.

### miR-142-5p-mediated Ulk1 repression controls dendritic formation underlying PHEV infection

**Figure 8:**
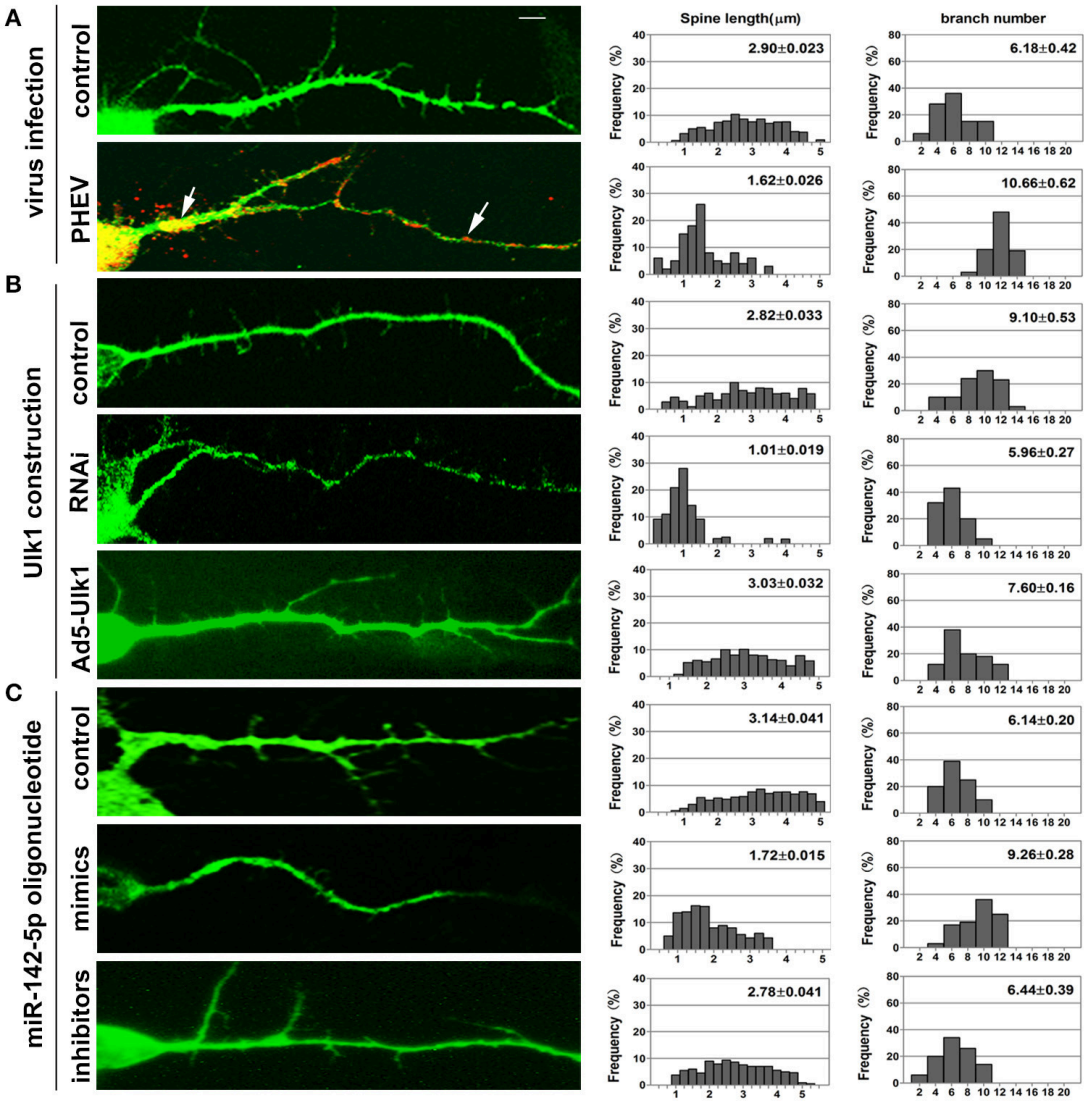
**Inhibition of Ulk1 blocks normal dendrites formation**. Primary cortical neurons (14 DIV) were incubated with the designated constructs or PHEV. At 16 DIV, neurons were stained with antibodies against MAP2 (green) or PHEV (red). Representative images were taken with the same magnification. Compared to control primary cortical neurons, the PHEV-infected **(A)**, Ulk1-RNAi-transfected **(B)**, or miR-142-5p mimics-treated neurons **(C)** showed stunted spine length, complex arborization and heterogeneous spine size. But, Ad5-Ulk1 and miR-142-5p inhibitor treatments made less significance. Notably, numerous irregular membrane blebs in PHEV-infected neurons gathered a large number of virus particles (arrows). Data represent the means of *n* = 50 cells per condition counted in triplicate ± s.d. In each histogram, the y-axis represents the percentage of neurons, and the x-axis represents the number of spines at certain lengths (the middle panel) or the number of branches (the right panel).

Neurons possess a soma, an axon and branched dendrites containing dendritic spines. To further define the physiological significance of PHEV infection in dendritic morphogenesis, we examined whether Ulk1 depression in mature 2- to 3-week-old neurons blocked spine formation. Without any treatment, normal spine formation occurs *in vitro* during the second and third weeks. Thus, we inoculated PHEV during this period and asked whether the virus inhibited endogenous Ulk1 and spine development. As shown in Figure 8A, control neurons showed robust spontaneous spine formation, but essentially no mature spines were observed in PHEV-infected neurons. And also, these PHEV-infected neurons exhibited stunted, bulbous spines, and numerous irregular membrane blebs, which gathered a large number of virus particles (Figure 8A). The virus blocked spinal stretch, but there was a modest increase in the number, which might due to self-repair capability after injury. To further investigate whether Ulk1 deficits regulate dendritic spinal volume in PHEV-infected neurons, Ulk1 siRNA and Ad5-Ulk1 were introduced to assess sequence-specific inhibition and overexpression of endogenous Ulk1 function. Naturally, dendrite spines in Ulk1 siRNA-transfected neurons were morphologically immature and similar to the spines seen in PHEV-infected neurons, however, the phenotype was opposite in the overexpression group (Figure 8B). After transfection of miR-142-5p mimics, the neurons showed monstrous morphology that was similar to virus-infected neurons at the same stage (Figure 8C). Taken together, Ulk1 plays a key role in the branched dendrite outgrowth and dendritic spine formation, and Ulk1 down-regulation by miR-142-5p is responsible for the nerve damage caused by PHEV.

### miR-142-5p antagomir blocked PHEV proliferation *In vivo*

**Figure 9:**
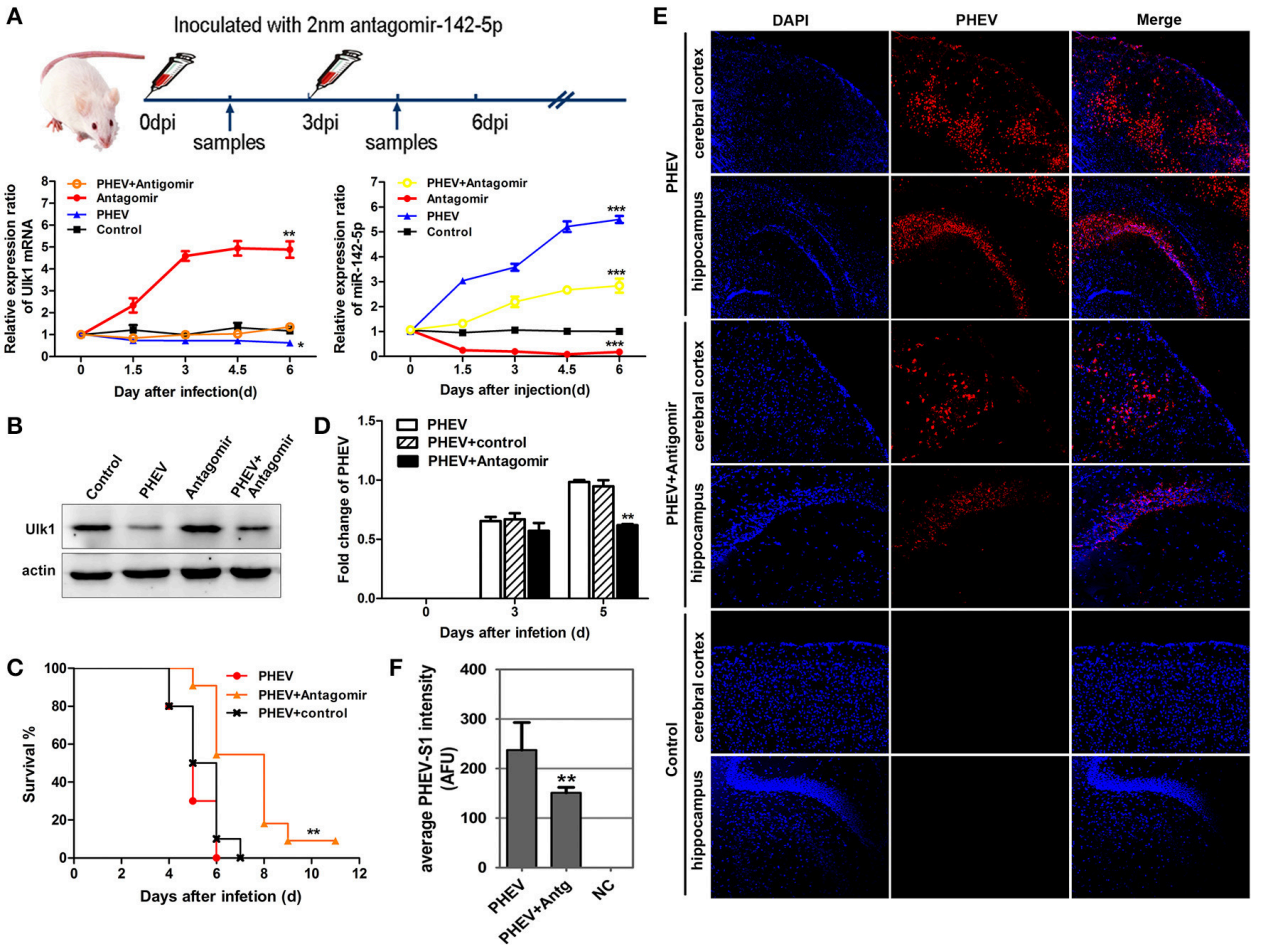
**miR-142-5p antagomir blocked PHEV proliferation in mice. (A)** Design and optimization of the appropriate treatment strategy for PHEV-infected mice. qRT-PCR showed that endogenous miR-142-5p was specially inhibited by antagomirs, and that Ulk1 expression was significantly enhanced. **(B)** The freshly isolated cerebral cortex lysate was harvested for Western blot at 6 dpi, and Ulk1 protein was enhanced after miR-142-5p treatment. **(C)** Mice were monitored daily for clinical signs, and survival curves are presented per time point. *n* = 10 mice per group for three independent experiments. **(D)** qRT-PCR of PHEV mRNA showed viral replication clearly blocked in the antagomir-treated mice. **(E)** Visualization of PHEV-infected cerebral cortexes and hippocampi from mice at 5 dpi. Upper panels show the mice incubated with PHEV alone, middle panels represent the antagomir-treated mice after PHEV infection and the lower panels represent the healthy mice. **(F)** Quantitative analyses of the PHEV staining intensity averaged (AFU) over the area showed statistically attenuated signals in the antagomir-treated mice. Error bars for all experiments represent the geometric means ± s.d. Asterisks indicate differences that were statistically significant (*P < 0.05, **P < 0.01, ***P < 0.001).

To determine if miR-142-5p functions in PHEV infection, miR-142-5p antagomirs, also known as anti-miRs or blockmirs are a class of chemically engineered and modified oligonucleotides that prevent other molecules from binding to a desired site on an mRNA molecule, was injected into the cerebral cortex of infected mice with a stereotaxic apparatus to silence endogenous microRNA. The effectiveness and specificity of the antagomir in inhibiting endogenous miR-142-5p expression and enhancing Ulk1 expression has been demonstrated in mice (Figure 9A). Compared with only PHEV infection group, miR-142-5p antagomir treatment significantly induced ulk1 protein expression in PHEV-infected or healthy mice at 6 dpi (Figure 9B). The survival times of the antagomir-treated mice were significantly extended compared to PHEV-infected or control-treated mice (Figure 9C), and they exhibited less prodrome (ruffled fur, hunched posture and standing walk), likely due to the lower miR-142-5p expression in the brain. At the same time, we found that PHEV proliferation was significantly restricted in the cerebral cortex of antagomir-treated mice at 5 dpi (Figure 9D). Immunofluorescence staining using PHEV mouse monoclonal antibodies showed that the number of positive PHEV-infected neurons was significantly reduced in the cerebral cortex and hippocampus of antagomir-treated mice (Figures 9E,F). Taken together, these results suggested a potential role for the miR-142-5p antagomir in blocking PHEV neurovirulence in the host, which prolonged the survival times and decreased the prodromal signs.

## Discussion

MicroRNAs are involved in post-transcriptional regulation, including development, differentiation, apoptosis, proliferation, and other processes (Bartel, 2009; Shukla et al., 2011). Over the past decade, numerous insights into the roles of microRNAs have greatly changed our conceptualization of the virus/host relationship (Friedman et al., 2009; Feldman and Tibbetts, 2015; Mizuguchi et al., 2015, 2016; Ruiz and Russell, 2015). For example, miR-27 was found to be degraded in latent Herpesvirus saimiri (HVS) infection, and as a destroyer caused T-lymphoproliferative disease in HVS-infected T cells (Chi et al., 2009). Instead, miR-122 exhibited a miRNA “sponge” effect and was sequestered by Hepatitis C virus (HCV) for long-term oncogenic potential (Machlin et al., 2011; Sedano and Sarnow, 2014; Luna et al., 2015). Although, abundant research has focused on virus-host microRNA interactions, the biological significance and underlying mechanisms have yet to be elucidated. More work in this area is needed.

PHEV, which primarily invades piglets and causes hemagglutinating encephalomyelitis, has a strong tropism for the CNS, where nerve cells but not glial cells are targets for viral replication (Hara et al., 2009), and lead to abundant microRNA up-regulation in mice. Of these microRNAs identified by microarray (data was not published), we found that miR-142-5p expression was strongly induced by PHEV over 8-fold at 5 dpi, and that altering miR-142-5p activity resulted in disordered neuronal morphogenesis in primary cortical neurons. Given that, some microRNAs have been reported to play crucial roles in CNS development, including axon guidance, dendritic morphogenesis, and synaptic plasticity (Wibrand et al., 2012; Sun et al., 2014; Liu et al., 2015; Ristori et al., 2015). Therefore, we attempted to explore the mechanism of nervous system dysfunction caused by PHEV from the perspective of the host's differentially expressed microRNA miR-142-5p.

Our data demonstrated that functional mRNA repression by miR-142-5p is an important mechanism for abnormal neuronal development in primary cortical neurons during PHEV infection, and that Ulk1 are required for axonal elongation and plasticity. Because of miR-122 antagonism has been in phase II clinical studies as an HCV therapy (Lanford et al., 2010; Janssen et al., 2013), which has shown that potently adjusting some microRNAs at a crucial level could lead to the rescue, or at least improvement, of nerve damage due to HCV. Thus, we presume that miR-142-5p antagonism is a potential novel strategy for PHEV therapeutic, which supported by antagonizing miR-142-5p resulted in PHEV replication restriction and prolonged survival times in PHEV-infected mice. However, the incompletely abolished viral replication indicated that there are other regulatory events in the physiological context of infection.

How might miR-142-5p molecules exert their functions? We performed deep sequencing and bioinformatics analyses, demonstrating that miR-142-5p could target the Ulk1 mRNA 3′UTR *in vitro* and *in vivo*. To gain insight into the function of the target gene, RNAi-mediated knockdown and adenovirus mediated overexpression of Ulk1 was performed, and we found that Ulk1 are required for axonal elongation and dendritic spines formation. This observation was consistent with a previous study that demonstrated that Unc51.1/Ulk1 was required for granule cell axon and dendrite spines formation (Tomoda et al., 1999, 2004). Generally, in the mammalian forebrain, the majority of excitatory synapses are located on dendritic spines, and the size of the spines correlates well with the strength of synaptic transmission (Paulin et al., 2016). Abnormal structural plasticity of excitatory synapses has also been strongly implicated in many neurodevelopmental, psychiatric, and neurodegenerative disorders (Woolfrey and Srivastava, 2016). Furthermore, co-localization of miR-142-5p and Ulk1 along axon shafts and/or within growth cones supported the functional involvement of these structures in modulating the neuron morphogenesis response to PHE. Consequently, we concluded that the abundance of miR-142-5p could repress Ulk1 mRNA, resulting in limited synthesis of new Ulk1 protein and restricted neuronal morphogenesis in underlying PHEV infection.

Post-transcriptional regulation of virus-induced host microRNAs significantly contributed to CNS dysfunction, which suggested that the combination of multiple microRNAs may have had therapeutic potential in viral nerve disease. Earlier research showed that Ulk1 is required for endocytosis in response to NGF/TrkA complexes (Pelkmans et al., 2005; Zhou et al., 2007; Lee and Tournier, 2011), and PHEV used the endo-/exocytosis for transsynaptic transfer (Li et al., 2013). In general, endocytosis and intracellular trafficking of NGF/TrkA complex is necessary for a successful NGF signal transduction process to facilitate neurite outgrowth and a hallmark of neuron differentiation (Zhang et al., 2000). In this paper, we found that miR-142-5p disrupts neuronal morphogenesis underlying PHEV infection by targeting Ulk1, whether the molecular mechanism is associated with endocytosis is still unknown. It was well known that NGF/TrkA complexes are endocytosed into Rab5a-positive early endosomes, and require inactivation of Rab5a to block early endosome fusion via RabGAP5 (Liu et al., 2007; Toda et al., 2008). Thus, we proposed that miR-142-5p-mediated Ulk1 repression acted locally at individual neutrites and/or intracellular membranous structures, which contributed to NGF signaling and transsynaptic communication of the neurotropic virus mainly via the vesicle-mediated secretory pathway. Future work is needed to obtain insights into the fate of the Ulk1-mediated pathway processes in regulating diverse downstream signaling events during PHEV infection. However, more work in this area is likely to be discovered.

## Author contributions

ZL and WH designed the experiments, supervised the experiments, and analyzed the data. ZL and YL performed experiments and interpreted the data. KZ, XL, ND, HL, JZ, HY, JS, and DS contributed reagents, materials and analysis tools. ZL, FG, and WH drafted the article or revised it critically for important intellectual content. All authors agree with final approval of the version for submission.

### Conflict of interest statement

The authors declare that the research was conducted in the absence of any commercial or financial relationships that could be construed as a potential conflict of interest.
