# Supplementary material for: miR-142-5p Disrupts Neuronal Morphogenesis Underlying Porcine Hemagglutinating Encephalomyelitis Virus Infection by Targeting Ulk1
Source: Front Cell Infect Microbiol. 2017 May 3;7:155. doi: 10.3389/fcimb.2017.00155 (PMC5413507; doi:10.3389/fcimb.2017.00155)
Supplement: Supplementary file 1 [file Table1.DOCX]

**Table S1. Sequences of all molecules used for the binding assays.**

| **Molecule** | **Sequence** |
| --- | --- |
| miR-142  anti-miR-142  anti-miR-142MIS  Ulk1-UTR  Ulk1-UTR MUT | 5’CAUAAAGUAGAAAGCACUACU 3’  5’A^*^G^*^T^*^AGTGCTTTCTACTTTA^*^T^*^G^*^ 3’  5’A^*^T^*^A^*^GTGGCTTTCTTTCATT^*^A^*^G^*^ 3’  5’GUAAGUCACCGGAGCACUUUAUG 3’  5’GUAAGUCACCGGAGCUGAAAUAG 3’ |

Sequences of the RNA and DNA molecules used in this study. For antisense oligodeoxynucleotides, phosphorothioate-modified bonds were designed according to the minimal modification criteria and indicated as *.
