# Supplementary material for: miR-142-5p Disrupts Neuronal Morphogenesis Underlying Porcine Hemagglutinating Encephalomyelitis Virus Infection by Targeting Ulk1
Source: Front Cell Infect Microbiol. 2017 May 3;7:155. doi: 10.3389/fcimb.2017.00155 (PMC5413507; doi:10.3389/fcimb.2017.00155)
Supplement: Supplementary file 2 [file Table2.DOCX]

**Table S2. Sequences of all molecules used for the RNAi assays.**

| **Molecule** | **Sequence** |
| --- | --- |
| Ulk1-RNAi | 5’-GATCCCCAGACTCCTGTGACACAGATTTCAAGAGAA  TCTGTGTCACAGGA GTCTTTTTTA-3’ |
| scrambled control | 5’-GATCCCCCATAGCATGCGTATCATGCTTCAAGAGAGC  ATGATACGCATGCTATGTTTTTA-3’ |

The hairpin sequences of the oligonucleotides were chosen for Ulk1 knockdown assays in primary cortical neurons.
